# Motivation to teach and preparedness for teaching among preservice teachers in China: The effect of conscientiousness and constructivist teaching beliefs

**DOI:** 10.3389/fpsyg.2023.1116321

**Published:** 2023-04-06

**Authors:** JiaLi Huang, Guoyuan Sang, Wenjie He

**Affiliations:** ^1^Center of Teacher Education Research, Beijing Normal University, Beijing, China; ^2^Institute of Curriculum and Instruction, Faculty of Education, Beijing Normal University, Beijing, China; ^3^College of Teacher Education, Capital Normal University, Beijing, China

**Keywords:** motivation to teach, constructivist teaching belief, conscientiousness, preparedness for teaching, pre-service teachers

## Abstract

“Preparedness for teaching” refers to the degree of confidence preservice teachers have, and reflects their ability. Developing preparedness for teaching is an important part of preservice teachers’ professionalization. A substantial body of literature has documented the critical influence of the motivation to teach on preparedness; however, how this relation is impacted by mediating and moderating mechanisms remains unclear. To respond to this gap in knowledge, the present study constructed a mediated moderation model through structural equation modeling and multigroup tests using 383 questionnaires completed by preservice teachers in China. The findings indicate that the preservice teachers’ genders, entry path, and levels of certainty about their future teaching career choices all influence their preparedness for teaching. Specifically, preservice teachers who believe that they will choose a teaching career in the future have more intrinsic motivation, stronger constructivist teaching beliefs, and a higher levels of teaching preparedness. Moreover, preservice teachers’ motivations to teach can positively predict their constructivist teaching beliefs and preparedness for teaching, but their constructivist teaching beliefs alone do not have a mediating effect on the relationship between motivation to teach and preparedness for teaching. However, the findings reveal that the constructivist teaching beliefs of highly conscientiousness group can partially mediate the relationship between the motivation to teach and the preparedness for teaching. Additionally, conscientiousness moderates the influence of constructivist teaching beliefs on preparedness for teaching. The study provides meaningful insights into the within-personal traits of how and when motivation to teach affects preparedness for teaching, which may be useful for the motivation best practices for preservice teacher recruitment, training, and support to create high-quality teachers.

## 1. Introduction

Keeping preservice teachers (PSTs) consistently and efficiently committed to their own learning has become key to producing high-quality teachers (Sinclair et al., 2006). In 2018, the government of mainland China enacted an action plan titled “The opinions of comprehensively deepening the reform of teacher construction in the new era,” which highlights the value and importance of high-quality teachers who are “happy to teach, suitable for teaching, and good at teaching.” Preparedness for teaching is often used as a learning outcome for PSTs—teachers with higher levels of preparedness are considered higher-quality teachers (Darling-Hammond et al., 2002; Stites et al., 2018; Van Rooij et al., 2019; Manowaluilou and Reeve, 2022). Based on Bandura’s (1977) social learning theory, PSTs’ preparedness for teaching can be affected by the long-term interaction between the environment and the individual. An individual’s perceptions and understanding of their environment are related to their will and ability to continue to commit to learning. People produce and execute actions based on their perceptions of their self-efficacy (Bandura, 1982); to some extent, an individual’s degree of confidence in their performance reflects their ability.

Notably, existing studies (e.g., Hechter, 2011; Lunenberg, 2011; Manowaluilou and Reeve, 2022) have confirmed that high self-efficacy influences engagement and performance and maintains self-development and self-adjustment among PSTs. Moreover, some studies have found that low self-efficacy impacts the decision to leave the teaching profession (e.g., Skaalvik and Skaalvik, 2007; Klassen and Chiu, 2011) and is moderately correlated with academic performance—indeed, it is negatively correlated with academic performance among high-achievers (e.g., Honicke and Broadbent, 2016; Talsma et al., 2019). Several studies (e.g., Tschannen-Moran and Hoy, 2001; Giallo and Little, 2003; Siwatu, 2007; Brown et al., 2015) have been conducted on the correlation between PST self-efficacy and preparedness for teaching, highlighting that preparedness positively affects teacher–student relationships, instructional strategies, and classroom management.

PSTs’ preparedness for teaching has not been sufficiently studied, despite its positive relationship to high-quality teacher (Hollins, 2011; Carter and Cowan, 2013). Several studies concern the sources (i.e., mastery experience, verbal persuasion, vicarious experience and physiological and affective states) that impact teacher self-efficacy (e.g., Poulou, 2007; Pfitzner-Eden, 2016; Clark and Newberry, 2019; Van Rooij et al., 2019) or the correlation or outcomes of self-efficacy (e.g., Oh, 2011; Jamil et al., 2012). Moreover, studies find that mastery experience impacts PSTs’ self-efficacy while verbal persuasion, vicarious experience, and physiological and affective states have smaller influence on PSTs (Pfitzner-Eden, 2016), and can predict but explain only 18% of preservice teachers’ preparedness for teaching (Clark and Newberry, 2019). Therefore, their preparedness must also be affected by other factors.

Regarding the development of PSTs, it is helpful to note that the psychological mechanisms for developing preparedness for teaching in situated teacher education programs remain a “black box” (Darling-Hammond, 2006). Drawing on social psychology, existing studies have used within-person designs to explain individuals’ performance and decisions across cultures and situations and over time (e.g., Schwartz, 2012; Schwartz et al., 2012; Vecchione et al., 2016). That is, PSTs’ psychological attributes (i.e., motivation to teach, personality, beliefs) provide the mechanisms for how they learn to become high-quality teachers (Rimm-Kaufman and Hamre, 2010). To date, few studies have simultaneously examined the psychological attributes (i.e., motivation to teach, personality, and beliefs) and preparedness for teaching of PSTs. For example, De Jong et al. (2013) find that PSTs’ personality (friendliness and extraversion) and self-efficacy appear not to be related to teacher-student relationships, while the relationships among personality, self-efficacy, and teacher-student relationship have not been explored. Meanwhile, Klassen and Tze (2014) indicate that teachers’ self-efficacy and personality contribute a significant but small effect size for teaching effectiveness, while motivation and personality are strongly linked with teaching efficacy (Poulou, 2007). Shrestha and Dangol (2020) assert that a positive relationship exists between conscientiousness and motivation among technical and vocational education teachers in Nepal, whereas the relationship of each factor and teachers’ performance is unknown. What roles do PSTs’ psychological attributes (namely: motivation, belief, and personality)—which underpin their persistence to pursue teaching and thus their active learning—play in their preparedness for teaching?

As a starting point for teacher preparation, the motivation to teach is widely situated as what drives PSTs to learn, and is thus mainly reflected in the motivation to receive teacher education (Sinclair, 2008; Torsney et al., 2019). Being motivated to teach implies that PSTs understand their own abilities, interests, ambitions, and limitations and the roles and responsibilities, conditions, requirements, and environments central to their careers (Brookhart and Freeman, 1992). Existing studies (e.g., Day et al., 2007; Bruinsma and Jansen, 2010; Chesnut and Burley, 2015) have found a strong positive relationship between motivation to teach and commitment to teaching. However, PSTs’ motivations to teach can change over time (Sinclair, 2008), and different motivations have different relationships with the commitment to teach (Zhang et al., 2019); Therefore, further research on how motivation to teach, which is related to other psychological attributes, affects preparedness for teaching is necessary.

Additionally, “beliefs”—as a psychosocial trait—indicate an individual’s real tendency to evaluate particular situations consciously or unconsciously and are a stable action factor; however, they can be changed (Borg, 2001). Notably, beliefs can influence the level of personal commitment to learning (Clark and Peterson, 1986; Ravindran et al., 2005). Research has shown that PSTs mostly hold a constructivist view of teaching and learning (Ogan-Bekiroglu and Akkoç, 2009; Cansiz and Cansiz, 2019) and can be predicted by mastery experience (Cansiz and Cansiz, 2019; Wang et al., 2022); while Chinese PSTs do not (Sang et al., 2009); however, their views can be changed over time due to their learning environment (McMinn et al., 2020; Li and Huang, 2023). How does constructivist belief related to other psychological attributes contribute to PSTs’ preparedness for teaching?

In addition, a growing body of studies (e.g., Bacanli, 2006; Bastian et al., 2017; Hartmann and Ertl, 2021) has confirmed that personality traits, as psychological qualities, represent consistent tendencies in PSTs’ actions and influence their career choices and willingness to continue teaching. Among the Big Five personality traits, conscientiousness is most directly related to motivation (McCrae and Costa, 1996) and most predictive of academic success (e.g., Furnham et al., 2003; Komarraju et al., 2009). However, how personality, as a stable element of psychological traits, especially conscientiousness affects PSTs’ learning to teach requires further investigation. For example, Oh (2011) asserts that personality, motivation, enactive mastery experience with social/verbal persuasion, and physiological/affective state can predict efficacy for classroom management, while how personality functions and what relationship among them are not explored. Jamil et al. (2012) indicate that there have had an association between teacher self-efficacy and observed performance, personality, and beliefs, though no causal inferences can be drawn.

Taken together, the above-mentioned studies inspire the following question: *What is the effect of constructivist teaching beliefs and conscientiousness on the relationship between motivation to teach and preparedness for teaching among PSTs in China?* The present study aimed to answer this question to uncover insights useful for determining how best to support the development of high-quality PSTs.

## 2. Conceptual framework

### 2.1. Preparedness for teaching

Preparedness for teaching is derived from Bandura’s concept of self-efficacy (Housego, 1990), which refers to people’s “beliefs in their capabilities to organize and execute the courses of action to produce given attainments” (Bandura, 1997, p. 3). Self-efficacy consists of efficacy expectations and outcome expectations. An efficacy expectation is “the conviction that one can successfully execute the behavior required to produce the outcomes”; meanwhile, an outcome expectation refers to “a person’s estimate [that] a given behavior will lead to [a] certain outcome” (Bandura, 1977, p. 193). An individual’s sense of their self-efficacy is notably self-referential; that is, people evaluate and alter their thinking and behavior (Bandura, 1977). Therefore, self-efficacy is a future-oriented belief about the level of competence individuals expect to demonstrate in a given situation (Tschannen-Moran and Hoy, 2001). Studies (e.g., Bandura, 1982; Pajares, 1996; Pajares, 2006) have verified that self-efficacy exceeds final performance as a predictor of future performance.

However, PSTs evaluate their efficacy that differed from in-service teachers (Evans and Tribble, 1986; Putman, 2012). A study by Woolfolk and Hoy (1990) situated teaching efficacy as comprising two factors: efficacy expectations regarding the extent to which teachers can perform their duties, and outcome expectations regarding the belief that teaching can influence student learning. Housego (1990) used efficacy expectations instead of self-efficacy to refer to PSTs’ perception of preparedness for teaching because PSTs did not believe their behaviors impacted student learning. As indicated by Tschannen-Moran and Hoy (2001) that until PSTs take responsibility for classroom teaching and management, their preparedness for teaching should be viewed as a holistic concept emphasizing more on efficacy expectations, and less on outcome expectations. Hence, we use “preparedness for teaching” to refer to PSTs’ perception of self-efficacy.

Drawing on Bandura’ social cognitive theory, Gibson and Dembo (1986) have constructed the concept of teacher efficacy with two components: personal teaching efficacy assuming that it reflected efficacy expectations, and teaching efficacy assuming that it reacted to outcome expectation. Housego (1990) concerns the development of student teachers’ feeling of preparedness for teaching in the classroom-teaching performance and student receptiveness during their teacher education year. Later, Tschannen-Moran and Hoy (2001) developed a reasonably valid and reliable measure, namely the Ohio State teacher efficacy scale (OSTES), to explore teacher efficacy that is composed of instructional strategies, student engagement, and classroom management. A large number of studies (e.g., Poulou, 2007; Klassen and Chiu, 2011; Putman, 2012; Van Rooij et al., 2019) have adopted the scale to conduct related research on PST’s teacher efficacy. Accordingly, we used these three components of PSTs’ self-efficacy to measure their preparedness for teaching.

### 2.2. Motivation to teach and preparedness for teaching

Drawing on Bandura (1977) self-efficacy theory, “motivation” is the cognitive source base of an individual’s capacity to imagine future consequences. Dörnyei and Ushioda (2011) define “motivation” as the direction and magnitude of human behavior. Meanwhile, “motivation to teach” refers to something that “attracts individuals to teaching” and impacts “how long they remain in their initial teacher education courses and subsequently the teaching profession, and the extent to which they engage with their courses and the teaching profession” (Sinclair, 2008, p. 37). Given that teaching has become a relatively unattractive career and the related trend of high rates of teacher attrition, existing research on teacher motivation has revealed that motivation to teach is a critical factor in attracting potential teachers to the profession and in encouraging PSTs to continually engage in professional development (Sinclair, 2008; Han and Yin, 2016).

Watt and Richardson (2007) Factors Influencing Teaching Choice (FIT-choice) scale presents 12 kinds of teacher motivations, such as intrinsic value, social utility value, and perceived teaching ability. Using the FIT-Choice scale to compare motivations to teach across the United States, Turkey, the People’s Republic of China, the Netherlands, Croatia, Germany, and Switzerland, they found that the similarities and differences in motivations to teach were related to differences in social and cultural values (Watt et al., 2012). Other scholars have similarly found that subgroups (such as elementary and secondary school educators) and cultural differences have also been related to differences in PSTs’ motivations to teach (Heinz, 2015). Generally, PSTs’ motivations to teach have been categorized into three types: intrinsic motives, extrinsic motives, and altruistic motives (e.g., Brookhart and Freeman, 1992; Thomson et al., 2012; Bergmark et al., 2018). Considering the traits of these three types reveals that PSTs primarily choose to go into teaching because it aligns with their altruistic, service-oriented goals and other intrinsic motivations—specifically, most teachers pursue their profession because they want to work with children and provide a service (Brookhart and Freeman, 1992). Additionally, the OECD (2005) concluded that the intrinsic benefits of teaching are related to intrinsic and altruistic motives and include working with children and adolescents and making a social contribution. Based on the suggestions of Brookhart and Freeman (1992) and the OECD (2005), the present study adopted the altruistic motive as an intrinsic motivation to refer to an individual’s sense of accomplishment and value due to the nature of the career (e.g., enjoying working up with children and service teaching) and figured extrinsic motivations as the external characteristics of teaching (e.g., stable job/pay, high social status) or incentives from others that encourage individuals to pursue a teaching career.

An individual’s desire to act to achieve a goal is positively related to the learning engagement of PSTs, and may also positively predict teaching efficacy (Jaengaksorn et al., 2015). Research has shown that the motivation to teach affects professional learning outcomes (König and Rothland, 2012) and commitment to teaching (Sinclair, 2008) among PSTs. The more PSTs understand that their motivation to teach comes from within, the better they may be able to overcome constraints in their environments and teach more effectively (Bruinsma and Jansen, 2010). In line with Bergmark et al. (2018), compulsory school PSTs (primary and middle) highlighted that their school’s caring mission and their intrinsic motives were the main reasons they chose teaching and for their success in their teacher training. In the first year of professional teaching, the interaction between PSTs’ motivation and their teaching efficacy predicted the reality shock expectation (Kim and Cho, 2014). Additionally, intrinsic motivation is more stable than extrinsic motivation in the learning journey of PSTs; however, although motivation is relatively stable, PSTs typically develop in a negative direction if their motivations change (e.g., they stop teaching; Sinclair et al., 2006). A similar finding was also reported by Bruinsma and Jansen (2010): PSTs with extrinsic maladaptive motives had negative teaching experiences and remained in the profession for shorter periods of time. These findings reveal that PSTs’ motivation to teach is the influencing factor in their preparedness for teaching. Therefore, we hypothesized that PSTs’ motivation to teach might positively affect their preparedness for teaching (H1).

### 2.3. The mediator of constructivist teaching beliefs

“Beliefs” are “psychologically-held understandings, premises or propositions about the world that are felt to be true” (Richardson, 1996, p. 104). “Teaching beliefs” refer to the perceptions and values that teachers hold about teaching, and they influence teachers’ views and practices about student learning, classroom management, and professional development, dominate teaching behaviors, and are more likely to influence teaching than the teachers’ professional knowledge (Clark and Peterson, 1986). Generally, based on the underlying theoretical orientation toward learning, which corresponds with transmissive/behaviorist or constructivist beliefs (Handal, 2003; Hassad, 2011), teaching beliefs are categorized into two types: traditional (i.e., a teacher-centered approach) and constructivist (i.e., a student-centered approach; Woolley et al., 2004; Berger and Lê Van, 2019). “Traditional teaching beliefs” mean that teachers believe that the aim of teaching is to transfer knowledge and that students are recipients of knowledge; meanwhile, “constructivist teaching beliefs” imply diverse and varied approaches to teaching, including problem-oriented learning, inquiry learning, and cooperative learning by which students construct their own comprehensive knowledge (Sang et al., 2010).

Studies have revealed that the teaching beliefs of PSTs directly affect many aspects of their learning to teach, such as their epistemologies of teaching strategies, student learning, and academic achievement (Ravindran et al., 2005; Voss and Kunter, 2020). A survey of Italian teachers suggested that self-efficacy can also be positively influenced when teachers hold conservative values, such as self-imposed limits, adherence to tradition, and emphasis on security and stability (Barni et al., 2019). Another study indicated that when PSTs hold teaching beliefs involving shallow and superficial rote memorization, their emphasis on competitive student performance can be positively predicted (Ravindran et al., 2005). Furthermore, PSTs who hold traditional teaching beliefs are confident about their classroom management and teaching strategies and their overall self-efficacy (Gürbüztürk and Sad, 2009).

Generally, in collectivist cultures, teachers may be more inclined to hold traditional teacher-centered beliefs and emphasize effective and fast face-to-face, direct teaching and controlled learning approaches. In contrast, in individualistic cultures, teachers tend to hold constructivist beliefs that are centered on student learning and may be more willing to spend time listening to students’ opinions, respect students’ choices, provide dialectical opportunities, and allow students to enjoy learning (Reeve et al., 2014). Of late, traditional teacher-centered beliefs are giving way to a more constructivist approach underpinned by the latest curriculum reform in China. According to the “Curriculum program and standards for compulsory education” issued in 2022, the main reform is rooted in constructivism; in particular, it emphasizes that students should actively build up competences and knowledge instead of transmitting directly from teachers (Tan, 2016). A body of studies has found that PSTs’ teaching beliefs are likely to change as they progress through a teacher education program, especially those regarding practice teaching (e.g., Sheridan, 2016) and personal (e.g., subject enjoyment, experience sharing) and social support (e.g., peers’ and mentors’ support) during their induction (Decker et al., 2015; Voss and Kunter, 2020). Therefore, this study used PSTs’ constructivist teaching beliefs developed throughout their teacher education programs in the context of China’s recent educational reform as a research variable.

Normally, the constructivist teaching beliefs of PSTs could predict their epistemology, such as the knowledge development associated with integrating of technology into teaching in China (Sang et al., 2010) and their cognitive engagement with their goals (Ravindran et al., 2005). PSTs who hold constructivist teaching beliefs are better able to understand the variability and complexity of student learning styles and are more willing to become proficient in various of teaching methods to improve their preparedness for teaching (Jamil et al., 2012). It has also been confirmed that PSTs are more likely to hold constructivist teaching beliefs that motivate them to become elementary school teachers (Heinz, 2015). In addition, a high motivation to teach is a positive predictor of the constructivist teaching beliefs of PSTs, and positively influences their preparedness for teaching (Voss and Kunter, 2020). Constructive teaching beliefs may mediate the relationship between motivation to teach and preparedness for teaching. Therefore, we hypothesized that constructive teaching beliefs are a positive mediating factor in the relationship between motivation to teach and preparedness for teaching (H2).

### 2.4. The moderator of conscientiousness

The OECD (2005) advised that there is a need to better understand the factors of PSTs’ educational success and entry into the profession. Studies on the pre-entry characteristics of PSTs identified that motivation, personality, and beliefs are predictive of their engagement and learning (e.g., Ravindran et al., 2005; Kim and Cho, 2014; Franz et al., 2022). Personality shapes individuals’ determination to pursue a particular career, cognitive capacity development, and educational attainment (Kankaraš, 2017). Several studies (e.g., Thornton et al., 2005; Eryilmaz, 2014) have also proven that a mature personality is key to the overall quality of preservice teachers and is also the core quality of teaching. “Personality traits” are stylistic and habitual patterns of affects, behaviors, and cognitions (Zillig et al., 2002; Jackson et al., 2010) and comprise “relatively enduring patterns of thoughts, feelings, and behaviours that reflect the tendency to respond in certain ways under certain circumstances” (Roberts, 2009, p. 140). Here, “patterns” and “relatively” mean that personality traits reflect a tendency to respond in certain ways in certain environments; along these lines, some aspects of personality may change in adulthood due to the influence of biological processes and needs (Roberts et al., 2006; Kawamoto, 2016). A study by Roberts (2006) based on social learning theory reported that the effect of environment on personality trait change was actually small; personality changes took a long time. The relatively fixed nature of personality has been verified as a moderator of susceptibility to environmental factors (Mertens et al., 2022). Meanwhile, scholars have also verified that personality does not predict PSTs’ desire to enter the teaching profession (Rockoff et al., 2011; Wiens and Ruday, 2014). A study by Perera et al. (2018) also indicated that models of teacher attrition, effectiveness, or selection should consider personality trait interactions instead of only the additive effects of personality; that is, personality may have a protective effect, as a moderator, on individuals’ behaviors.

In terms of PSTs, those who want to and do become teachers belong to a special group in terms of personality (Thornton et al., 2005). The Big Five traits, a widely used instrument for assessing personality, comprise five personality traits; namely: neuroticism (i.e., negative emotion, anxiety, and low self-esteem), extraversion (i.e., sociable and assertive), openness (i.e., curious and imaginative), agreeableness (i.e., sympathetic and easily moved), and conscientiousness (i.e., a high degree of responsibility and determination; Costa and McCrae, 1992; Ripski et al., 2011). A large number of studies have highlighted the importance of the relationship between PSTs’ personality traits and their performance, self-efficacy, and willingness to keep teaching. For example, a study by Franz et al. (2022) indicated that extraversion and neuroticism were crucial personality traits as PSTs seem to be rather homogeneous in terms of the other traits. Meanwhile, other studies have explored PSTs’ performance by connecting their cognitive abilities with different personality traits. Ripski et al. (2011) found that extraversion may change significantly during PSTs’ time in a teacher education program and benefit from their relationships with students. Wiens and Ruday (2014) examined the connection between teaching performance, feelings about teaching, and personality, and found that PSTs were highly agreeable and conscientious, which helped them achieve academic success. These findings are notably not consistent. However, “conscientiousness” refers to the willingness to follow the rules and to exert effort, which could be best viewed as a measure of trait-oriented work motivation (i.e., willingness to do; Schmidt and Hunter, 1998). Additionally, among the five personality traits, conscientiousness is most closely aligned with the characteristics expected of teachers in Chinese society (Li and Ye, 2009). Therefore, we studied the effect of conscientiousness on PSTs’ preparedness for teaching in China.

“Conscientiousness” is characterized as the degree to which an individual’s responsibility, order, impulse control, and laziness persistently and steadily influence their behavior (Jackson et al., 2010). Existing studies have reported conflicting findings about the effects of conscientiousness. For example, Djigić et al. (2014), Baier et al. (2019), and Aydın et al. (2013) found that conscientiousness is an effective predictor of teaching efficacy, teaching enthusiasm, and classroom management. Meanwhile, a study by Shrestha and Dangol (2020) showed that highly conscientious vocational education teachers demonstrate high job performance, achieved through high levels of compliance and hard work. However, a study by Bastian et al. (2017) reported that different levels of conscientiousness might have different effects, and that conscientiousness may have a moderating effect on PSTs’ preparedness for teaching. The “well-adjusted” latent profile of teachers’ tendencies was also verified by moderately high levels of extraversion, openness, agreeableness, and conscientiousness (Perera et al., 2018). Given that the above-mentioned studies treat personality as a moderating variable (e.g., Barrick and Mount, 2005; Rockoff et al., 2011; Wiens and Ruday, 2014; Perera et al., 2018), we hypothesized that conscientiousness plays a moderating role in the relationship between motivation to teach, constructivist teaching beliefs, and preparedness for teaching in China (H3).

**Figure 1:**
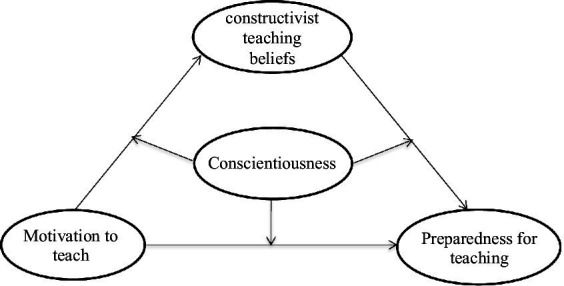
A concept model of the mediated moderation.

This study aims to expand the knowledge on the relationship of PSTs’ psychological attributes with their preparedness for teaching, as it is unclear how and when the motivation to teach affects preparedness to teach. Hence, we treat PSTs’ motivation to teach as the driving force behind their career choice, constructivist teaching beliefs as playing a mediating role, and conscientiousness as playing a moderating role to establish a mediated moderation model (see Figure 1).

## 3. Data and methods

### 3.1. Participants

Cluster sampling was employed to recruit a total of 400 master’s degree students (from the class of 2020) to complete a questionnaire. During teacher’s college, the participants majored in 15 different academic disciplines, including mathematics, English, Chinese language and literature, and physics. After excluding invalid questionnaires, 383 valid questionnaires were obtained, for an effective recovery rate of 95.75%. The subjects had a mean age of 24.20 years, with a standard deviation (SD) of 1.65 years.

Written informed consent was gathered from participants. The participants were advised of the purpose of the study and told that all data would be kept confidential and only used by the researchers for the purpose of the study. The participants were also informed that their participation was voluntary and that they could write down their feelings and thoughts. They completed a paper-and-pencil questionnaire within 25–30 min in their classroom.

### 3.2. Measures

The “Preservice Teacher Personality and Readiness to Teach Scale” (written in Chinese) was created for this study. It includes four subscales, the Big Five personality dimensions, motivation to teach, constructivist teaching beliefs, and preparedness for teaching. In addition to detailing their demographic characteristics, the participants were asked to answer questions using 6-point Likert scales ranging from 1 (strongly disagree) to 6 (strongly agree). The following subscales were used to evaluate different variables.

#### 3.2.1. Preparedness for teaching

The scale for preparedness for teaching was taken from the self-efficacy subscale of the Teaching and Learning International Survey (TALIS; OECD, 2019). The scale consisted of three dimensions; namely: instructional strategies, classroom management, and student engagement, with a total of 12 items. Among them, preparedness for instruction was measured using six items (e.g., teaching in a way that students can understand the content of the subject, explaining by giving different examples when students feel confused); preparedness for classroom management was measured using three items (e.g., keeping students disciplined in class and calming down hyperactive students); and preparedness for student engagement was measured by three items (e.g., clearly expressing the expectations for student behavior regarding helping students to recognize the value of learning). The mean scores for all items were combined, and the higher the total score, the more the individual’s situation was consistent with the description and the higher their preparedness for teaching. Confirmatory factor analysis (CFA) showed that the three-factor model of the scale for assessing preparedness for teaching had a mediocre fit (Hooper et al., 2008) with *χ*^2^/df = 4.732, the Tucker-Lewis index (TLI) = 0.88, the comparative fit index (CFI) = 0.90, the goodness of fit index (GFI) = 0.86, and root-mean-square error of approximation (RMSEA) = 0.09. Cronbach’s α was 0.92.

#### 3.2.2. Motivation to teach

The subscale for motivation to teach combined items from TALIS’s (OECD, 2019) motivation subscale to measure intrinsic and extrinsic motivation, with a total of seven items. Specifically, there were four items for intrinsic motivation (e.g., “I like teaching,” “I want a job spending time with kids and teenagers,” “I am very interested in teaching a particular subject”). Extrinsic motivation consisted of three items (e.g., “job stability,” “winter and summer breaks for teachers,” and “others think I am fit to be a teacher”). The mean scores of all the questions were combined, and the higher the total score, the stronger the PSTs’ motivation to become teachers. The CFA indicated that the scale had a mediocre fit (Hooper et al., 2008), with *χ*^2^/df = 4.38, TLI = 0.88, CFI = 0.92, GFI = 0.95, and RMSEA = 0.09. The Cronbach’s α for the internal consistency of the scale was 0.81.

#### 3.2.3. Constructivist teaching beliefs

This scale was adopted from TALIS (OECD, 2019) subscale and consisted of four items: “The teacher’s role is to help students to explore,” “The best way to learn is for the students to solve the problems themselves,” “The teacher should allow students to solve a problem before offering the solution to the problem,” and “The process of thinking and reasoning is more important than specific course content.” Cronbach’s α was 0.72, composite reliability (CR) was 0.83 (>0.7), and the average variance extracted (AVE) was 0.551 (>0.5), showing acceptable reliability and validity.

#### 3.2.4. Conscientiousness

This scale was derived from the Short Version of the Chinese Adjectives Scale of Big Five Factor Personality (BFFP-CAS-S) developed by Luo and Dai (2018). We used Chinese bipolar adjectives as test items and adopted a 6-point Likert scoring system. In the present study, the four conscientiousness items in the BFFP-CAS-S were adopted; Cronbach’s α was 0.733, CR was 0.83 (>0.7), and AVE was 0.558 (>0.5), showing acceptable reliability and validity.

### 3.3. Data analysis

We used a Maximum Likelihood estimation for the latent variable model evaluation with IBM SPSS version 22 and Amos 22.0. First, an analysis of variance (ANOVA) was used to analyze variations in the demographic characteristics of the PSTs to uncover the impacts of gender, undergraduate major, entry path, full-time teaching experience, and choice to pursue a teaching career on motivation to teach, constructivist teaching beliefs, and preparedness for teaching.

Considering the inclusion of measurement error, structural equation modeling (SEM) was conducted to analyze the presence, direction, and strength of relations between latent variables representing constructs. Because chi-squared values are sensitive to sample size, *χ*^2^/df less than 3 was used as the fit criterion (Schreiber et al., 2006). In addition, CFI (>0.90), TLI (>0.90), SRMR (<0.08), and RMSEA (<0.06) were used as fitting indices (Hu and Bentler, 1999). The bootstrap technique was used to test the mediating effect, and zero was not included in the 95% confidence interval (CI) (Shrout and Bolger, 2002).

A multigroup SEM was constructed to test the moderating effect. As suggested by Byrne (2006, p. 255), “to ensure meaningful and credible interpretation of the structural paths, it is important to know that the measurement parameters are operating in the same way for both groups under study.” Therefore, the baseline model of best fit for each group separately was first tested to obtain the Chi-squared values (*χ*2unre) for all paths estimated individually; then, the two groups of paths were restricted to the same restricted model to obtain the Chi-squared values (*χ*2re) for all paths, and the baseline model and the restricted model were combined to form a nested model for statistical testing. If the Δ*χ*^2^ of the baseline model is significant (*p* < 0.05), the restricted model has a poor goodness of fit and therefore the hypothesis that the coefficients of the paths are the same is rejected, indicating that the existence of a moderating effect is supported. Conversely, if the Δ*χ*^2^ is not significant (*p* > 0.05), the existence of the moderating effect is not supported (Hair et al., 2010).

## 4. Results

The ANOVA results in Table 1 indicate that, except for student engagement, the differences in the preparedness for teaching of PSTs of different genders were significant (*p* < 0.05); specifically, compared with female students, male students were more confident in their preparedness for teaching. The differences in the constructivist teaching beliefs and student engagement efficacy of PSTs with different entry paths were also significant, with negative *t*-values (*p* < 0.05), and indicated that PSTs who were admitted into graduate programs for which entrance examinations were waived held more constructivist teaching beliefs and were more confident in their efficacy of student engagement. Meanwhile, the differences in the classroom management efficacy of PSTs with or without full-time teaching experience were significant (*p* < 0.05); in particular, PSTs with full-time teaching experience were more confident in their classroom management skills than those without such experience. In addition, the *t*-values for the variables of motivation to teach, constructivist teaching beliefs, and preparedness for teaching were not significant (*p* > 0.05) regarding whether PSTs majored in teacher education as undergraduate students, indicating that their scores for each variable did not differ depending on whether or not they majored in teacher education at the undergraduate level.

**Table 1:** Demographic characteristics of preservice teachers and score differences for various variables.

Basic characteristics	Number of people	Motivation to teach	Intrinsic motivation	Extrinsic motivation	Teaching beliefs	Preparedness for teaching	Teaching	Classroom management	Student engagement
		M ± SD	M ± SD	M ± SD	M ± SD	M ± SD	M ± SD	M ± SD	M ± SD
**Gender**									
(1) Male	34	3.16 ± 0.87	4.03 ± 0.57	2.28 ± 0.42	3.28 ± 0.49	3.92 ± 0.59	3.53 ± 0.53	2.89 ± 0.53	2.38 ± 0.36
(2) Female	349	3.19 ± 0.77	4.02 ± 0.58	2.37 ± 0.39	3.39 ± 0.44	3.72 ± 0.56	3.16 ± 0.51	2.57 ± 0.54	2.64 ± 0.35
*t* value		−0.568	0.77	−1.23	−1.38	3.88	4.02	3.30	1.85
*p* value		0.571	0.939	0.219	0.168	0.000^***^	0.000^***^	0.001^**^	0.065
Cohen’s value		0.10			0.24	0.68^***^			
**Entry path**									
(1) Entrance examination	347	3.18 ± 0.39	4.01 ± 0.57	2.35 ± 0.40	3.37 ± 0.45	3.74 ± 0.57	3.18 ± 0.52	2.59 ± 0.54	2.62 ± 0.35
(2) Entrance examination waived	36	3.30 ± 0.40	4.13 ± 0.59	2.50 ± 0.30	3.52 ± 0.35	3.92 ± 0.60	3.33 ± 0.52	2.69 ± 0.58	2.40 ± 0.38
*t* value		−1.67	−1.18	−1.57	−2.0	−1.79	−1.63	−1.08	−2.19
*p* value		0.10	0.24	0.12	0.046^*^	0.075	0.104	0.283	0.029^*^
**Choosing a teaching career in the future**									
(1) Yes	284	3.23 ± 0.39	4.09 ± 0.58	2.36 ± 0.39	3.41 ± 0.43	3.74 ± 0.56	3.18 ± 0.51	2.58 ± 0.54	2.28 ± 0.36
(2) No^1^	1	-	-	-	-	-	-	-	-
(3) Not sure	98	3.10 ± 0.37	3.84 ± 0.51	2.36 ± 0.42	3.29 ± 0.47	3.81 ± 0.60	3.24 ± 0.55	2.67 ± 0.55	2.27 ± 0.35
*t* value		2.72	3.72	0.02	2.49	−1.04	−1.04	−1.35	0.07
*p* value		0.007^**^	0.000^***^	0.986	0.013^*^	0.298	0.298	0.179	0.941

(1) There is only one person; therefore, the data are included in the case (3) calculation. (2) *p < 0.05, ***p < 0.001.

Overall, PSTs who wanted to pursue teaching had relatively strong motivations to teach and intrinsic motivations, and most held constructivist teaching beliefs and were confident in their student engagement skills. PSTs with full-time teaching experience were notably more confident in their classroom management skills.

As Table 2 makes clear, our preliminary analyses revealed that the scales were reliable and offered basic data for SEM. As the variables in the study were self-reported, Harman’s single-factor test was used to examine whether the results were affected by a common method bias. In addition to CFA, which rejected the single-factor model (*χ*^2^ = 2253.313, *df* = 350, NFI = 0.593, IFI = 0.633, CFI = 0.631, TLI = 0.601, RMSEA = 0.119), an exploratory factor analysis using SPSS 22.0 was also conducted to run this test. For the exploratory factor analysis, the unrotated factor solution showed that a single factor could account for only 32.318% of the total variance (<40.0%). Therefore, the single-factor model was rejected by both the confirmatory and exploratory factor analyses, indicating that common method variance did not impair the results.

**Table 2:** Correlations among the variables.

Item	1	2	3
1. Motivation to teach	1		
2. Preparedness for teaching	0.357**	1	
3. Constructivist beliefs	0.203**	0.211**	1
4. Conscientiousness	0.050	0.097	−0.081

**p < 0.01.

**Figure 2:**
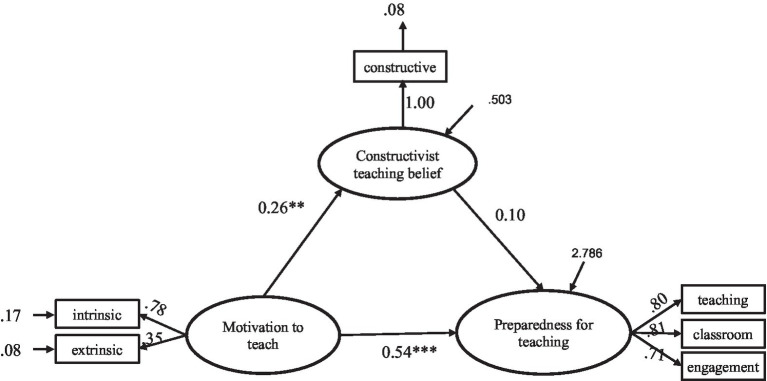
The structural equation model.

An SEM model was constructed to analyze how the motivation to teach and constructivist teaching beliefs influence PSTs’ preparedness for teaching. The main variables were first standardized to reduce multicollinearity and improve the convergence of the model. The structural equation model test results showed a good fit (Figure 2): *χ*^2^/*df* = 1.743 (<3), *p* < 0.001, CFI = 0.991 (>0.95), TLI = 0.98 (>0.95), RMSEA = 0.044 (<0.06), and SRMR = 0.0232 (<0.08).

The model was further tested; the results are provided in Table 3. Regarding direct effects, the direct effect of motivation to teach on constructivist teaching beliefs was significant (*β* = 0.264, *p* < 0.01). The direct effect of constructivist teaching beliefs on preparedness for teaching was not significant (*β* = 0.105, *p* > 0.05). The direct effect of motivation to teach on preparedness for teaching was significant (*β* = 0.537, *p* < 0.001), indicating H1 was supported. To test the mediating role of constructivist teaching beliefs in the relationship between motivation to teach and preparedness for teaching, zero was included in both the unstandardized 95% CI of [−0.027, 0.171] (*p* = 0.076) and the standardized 95% CI of [−0.018, 0.076] (*p* = 0.150) after 2,000 bootstrap replications, indicating that constructivist teaching beliefs did not mediate this relationship, which did not support H2 (see Table 4).

**Table 3:** Multigroup analysis: Testing for path coefficients invariance across high and low score group conscientiousness (*N* = 383).

Structural model	*χ*2	df	Δ*χ*2	Δdf	*χ*2/df	*p*	CFI	TLI	RMSEA	SRMR
Model 1 Baseline model	18.72	14	-	-	1.34	0.18	0.99	0.98	0.03	0.03
Model 2 Restricted model	27.21	17	8.49	3	1.60	0.05	0.98	0.97	0.04	0.03
Model 3 Motivation to teach → Constructivist beliefs	18.91	15	0.19	1	1.26	0.22	0.99	0.99	0.03	0.03
Model 4 Constructivist beliefs → Preparedness for teaching	25.26	15	6.54^*^	1	1.68	0.047	0.98	0.96	0.04	0.06
Model 5 Motivation to teach → Preparedness for teaching	19.74	15	1.04	1	1.32	0.18	0.99	0.98	0.03	0.06

*p < 0.05.

**Table 4:** Structural equation model path estimation.

Path relationship	*B*	*SE*	*t* value	*β*
**Direct effect**				
Motivation to teach → Constructivist belief (a)	0.258	0.126	2.9^**^	0.264
Constructivist beliefs → Preparedness for teaching (b)	0.199	0.199	1.611	0.105
Motivation to teach → Preparedness for teaching (c)	1.00	0.492	3.35^***^	0.537
**Indirect effect**				
a × b	0.051	0.034	1.408	0.028

**p < 0.01, ***p < 0.001.

**Figure 3:**
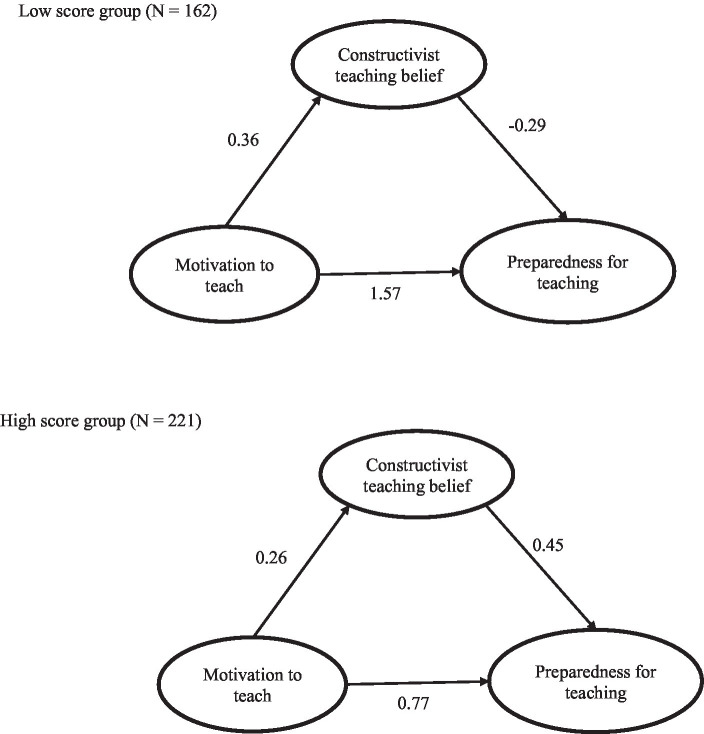
SEM for the low and high conscientiousness score.

To test the moderating effect of conscientiousness, the participants were divided into a high-score group (*n* = 221) and a low-score group (*n* = 162) using the mean value obtained from the conscientiousness scale. The goodness-in-fit indices for the SEM run with low and high score groups are provided in Figure 3. SEM results for the low score group showed a goodness in fit: *χ*^2^/*df* = 0.95 (<3), *p* < 0.001, CFI = 1.00 (>0.95), TLI = 1.00 (>0.95), SRMR = 0.03 (<0.08) and RMSEA = 0.00 (<0.06). Results for the high score group reported the same goodness in fit to the data: *χ*2/*df* = 1.73 (<3), *p* < 0.001, CFI = 0.99 (>0.95), TLI = 0.97 (>0.95), SRMR = 0.03 (<0.08) and RMSEA = 0.058 (<0.06). As seen in Table 3, the evidence by a significant chi-square difference (Δ*χ*^2^ = 8.49, *df* = 3, *p* = 0.05) indicates that there were marginally significant differences in path estimates of low versus high score groups between the baseline model (model 1) and the restricted model (model 2). Additionally, the chi-square difference was different among paths. The paths from motivation to teach to constructivist beliefs (Δχ^2^ = 0.19, *df* = 1, *p* = 0.22) and motivation to teach to preparedness for teaching (Δχ^2^ = 1.04, *df* = 1, *p* = 0.18) did not differ significantly between the two groups. However, the paths from constructivist beliefs to preparedness for teaching (Δ*χ*^2^ = 6.54, *df* = 1, *p* < 0.05) was significantly higher in the group that scored high on the conscientiousness subscale than in the low group. Overall, H3 was supported. These findings reveal that the conscientiousness of PSTs plays a moderating role in the influence of constructivist teaching beliefs on preparedness for teaching. Thus, highly conscientious PSTs with relatively strong constructivist teaching beliefs are likely to demonstrate relatively high levels of preparedness for teaching; conversely, PSTs that are not very conscientious with relatively strong constructivist teaching beliefs are likely to demonstrate relatively low levels of preparedness for teaching.

## 5. Discussion

The present study provides several essential findings that further knowledge of the effects of PSTs’ within-person factors on their preparedness for teaching, especially in the context of China. More specifically, motivation to teach can be a powerful factor in PSTs’ preparedness for teaching and constructivist teaching beliefs. We found that constructivist teaching beliefs alone did not mediate between motivation to teach and preparedness for teaching. Moreover, the conscientiousness of PSTs was an interfering factor in the second half of the intermediary model (i.e., constructivist teaching belief and preparedness for teaching).

### 5.1. Differences in demographic characteristics

First, the results of the present study showed that among the participants, male students were more confident in their preparedness for teaching than female students; this supports some previous research (e.g., Klassen and Chiu, 2010). Across careers, male students are usually more confident in their abilities, while females usually have low self-confidence, even if they have similar abilities to males (Bandura et al., 2001). In addition, participants with teaching experience exhibited confidence in classroom management and preparedness for teaching, consistent with previous findings (e.g., Vaudroz et al., 2015).

Furthermore, the present study found that, among the participants, PSTs who entered graduate programs in teacher education for which entrance examinations were waived were more confident regarding student engagement and had stronger constructivist teaching beliefs than those who entered programs with entrance examinations. Similar to many previous studies (e.g., Sinclair, 2008; Jaengaksorn et al., 2015), we indicate that the potential role of motivation to teach in becoming a quality teacher among PSTs should not be underestimated, especially in China. PSTs who planned to become teachers exhibited a motivation to teach, intrinsic motivation, and constructivist teaching beliefs. Moreover, the results of the present study were similar to those reported by Zhang et al. (2013), confirming that some characteristics of the teaching career itself, such as promoting socialization and society’s love for children, attract preservice teachers to teaching. For PSTs who are admitted to graduate programs, priority should be given to those who “will choose a teaching career in the future,” and the number of graduate students enrolled in programs that waive entrance examinations should be increased.

### 5.2. The relationship between motivation to teach, constructivist teaching beliefs, and preparedness for teaching

Second, the results of the present study, in line with previous studies (e.g., Jaengaksorn et al., 2015; Lysaght et al., 2018) indicated that motivation to teach positively impacts on both constructivist teaching beliefs and preparedness for teaching. Notably, motivation to teach had a greater direct influence on preparedness for teaching (*β* = 0.54, *p* < 0.001) than constructivist teaching beliefs (*β* = 0.26, *p* < 0.01), indicating that the potential role of motivation to teach in PSTs becoming quality teachers should not be underestimated. Motivation to teach varies depending on the sociocultural environment (Watt et al., 2012). The results of the present study are highly similar to those in Watt et al. (2012) study, which was conducted in different sociocultural contexts (e.g., Australia, the United States, Norway, and Germany) as well as to the results of a study by Guo and Sun (2018). Taken together, these findings confirm that the internal characteristics of the teaching career, such as serving society, giving back to society, enjoying teaching, and helping students grow, enhance the positive perceptions of PSTs and consistently drive effective learning.

### 5.3. Testing the moderation model

Unexpectedly, we found that PSTs’ constructivist teaching beliefs did not directly influence preparedness for teaching and did not mediate the relationship between motivation to teach and preparedness for teaching. This unexpected finding differs from the results of some previous studies (e.g., Jamil et al., 2012). However, our finding is aligns with Baier et al. (2019) study which indicated that, in terms of personality, constructive beliefs might be a less important predictor of teacher effectiveness. The result was also consistent with Dong et al. (2015) study, which found that a strong belief in constructivist teaching positively impacts student engagement and learning but does not predict PSTs’ technological pedagogical content knowledge.

Based on the potential impact of the environment, established by social learning theory, constructivist beliefs are influenced both by the individual’s subjective interpretation of their active experience and their interactions with others (Mascolo and Fischer, 2004). Teachers’ beliefs are most profoundly influenced by their own long-term educational experiences as students, subject knowledge, and social cultures (Kagan, 1992; Pajares, 1996; Richardson, 1996). Scholars have previously suggested that PSTs’ teaching beliefs are relatively stable (e.g., Darling-Hammond, 2006; Korthagen et al., 2006; Vidović and Domović, 2019). Ye et al. (2022) reported that Chinese PSTs who cared for students and acted responsibly while teaching were immersed in learning a kind of teacher morality through social interactions. Additionally, Levin (2015) indicated that teaching beliefs about moral and ethical dilemmas and societal issues affect teaching (e.g., politics, poverty, economics). Notably, teachers generally accept existing sociocultural beliefs (Kaur and Noman, 2015), which the present study inferred as possibly related to PSTs’ long-term immersion in traditional education, therefore may play a role in stabilizing belief systems in China. Along these lines, an existing study reported that Chinese teachers tend to hold traditional teaching beliefs (Sang et al., 2009) and another indicated that teachers in collectivist cultures mostly ascribe to traditional teacher-centered pedagogies (Reeve et al., 2014). Under the prevailing sociocultural belief that teacher-centered pedagogy is the most effective and efficient approach to advancing student learning outcomes, PSTs understand that Chinese teachers bear a professional responsibility to promote student growth and development, and are influenced by the traditional beliefs of teaching to the test and teacher-centeredness that allow PSTs to perceive the limitations of their environments. This perception causes PSTs to lower their constructivist teaching beliefs and thus choose to adapt to their environments.

From our unexpected finding, the present study further found that the constructivist teaching beliefs of highly conscientious PSTs partially mediate the relationship between their motivation to teach and their preparedness for teaching. Furthermore, conscientiousness, in its relationship with motivation to teach, constructivist teaching beliefs, and preparedness for teaching, has a moderating effect on the second half of the intermediary model (i.e., the path from constructivist teaching beliefs to preparedness for teaching); that is, among highly conscientious PSTs, the stronger their constructivist teaching beliefs, the higher their preparedness for teaching; conversely, among PSTs that are not very conscientiousness, the stronger their constructivist teaching beliefs, the weaker their preparedness for teaching.

Conscientiousness is closely related to some professional characteristics, such as efficacy, goal setting, and overcoming obstacles (Wendling and Sagas, 2020). A personality trait that includes persevering and doing one’s best, conscientiousness has been shown to improve teaching efficacy (Bayona and Castañeda, 2017). Regarding conscientiousness, this study deepened the findings of Sang et al. (2009), who showed that under the influence of Confucian culture, collective consciousness has challenged the constructivist teaching beliefs of PSTs in China. Specifically, this study indicated that conscientiousness, as a personality trait characterized by responsibility and loyalty, allows PSTs to overcome their intrinsic conflicts and positively influences their preparedness for teaching. This finding may be explained by the facts that coping and defense mechanisms may help people to reconfigure information in a way that inoculates them from the necessity to change and that individuals who can take responsibility for themselves will engage in habits that will enable them to attain their goals (Roberts, 2009). When coupled with high conscientiousness, in terms of understanding teacher duties and the sense of responsibility for students’ learning, constructivist teaching beliefs can even enhance the positive impact of motivation to teach on preparedness for teaching.

## 6. Conclusion

The present study is one of only a few studies to consider the impact of motivation to teach, constructivist teaching beliefs, and conscientiousness on PSTs’ preparedness for teaching. Drawing on social learning theory, most previous studies have focused on how the interaction effects between the contextual and personal factors of PSTs influence their preparedness for teaching (e.g., Clark and Newberry, 2019). In the present study, we investigated within-personal traits of PSTs that affect the relationship between the motivation to teach and preparedness for teaching and explored the mediated moderation effect of conscientiousness and constructivist teaching beliefs in the Chinese context. Our research filled a gap in social learning theory to explain the psychological attributes that come from the individuals’ responses to the environment. This study notably confirmed and deepened the findings of previous studies on the positive effects of motivation to teach (e.g., Sinclair, 2008; Jaengaksorn et al., 2015); specifically, this study showed that PSTs’ motivation to teach could be strengthened by constructivist teaching beliefs that align with the spirit of existing educational reforms in China. However, this study also unexpectedly revealed that constructivist teaching beliefs do not facilitate PSTs’ preparedness, indicating that PSTs may have internalized Chinese traditional culture. Additionally, conscientiousness played a protective role in promoting the moderating effect on the relationship between constructivist teaching beliefs and preparedness for teaching, indicating that the personalities of PSTs should be considered when teaching PSTs constructivist teaching beliefs to facilitate their preparedness for teaching.

This study had some limitations and, therefore, can inspire some directions for future research. The results of the present study highlight the moderating effect of PSTs’ conscientiousness on the relationship between constructivist teaching beliefs and preparedness for teaching; however, this study was limited in its focus on context- and personality-related factors, with conscientiousness selected due to the unique social context in China. Given that teachers’ roles, responsibilities, and social norms often differ across countries, much more research is necessary to understand the effect of conscientiousness and its place in the process of PSTs’ teacher education, including recruitment, training, and retention, across countries. Moreover, the unexpected effect of constructive teaching beliefs opens up new questions about what mechanisms can change or maintain beliefs (Decker et al., 2015; Sheridan, 2016; Voss and Kunter, 2020); this should be the subject of further investigation.

## Data availability statement

The raw data supporting the conclusions of this article will be made available by the authors, without undue reservation.

## Author contributions

JH, GS, and WH contributed to conception and design of the study. WH organized the database. JH and GS performed the statistical analysis. JH wrote the first draft of the manuscript. JH, GS, and WH wrote sections of the manuscript. All authors contributed to the article and approved the submitted version.

## Funding

This work was funded by the Fist-class Discipline Training Action Program, Beijing Normal University (YLXKPY-ZYSB202203) and the International Joint Research Project of Huiyan International College, Faculty of Education, Beijing Normal University (ICER202001).

## Conflict of interest

The authors declare that the research was conducted in the absence of any commercial or financial relationships that could be construed as a potential conflict of interest.

## Publisher’s note

All claims expressed in this article are solely those of the authors and do not necessarily represent those of their affiliated organizations, or those of the publisher, the editors and the reviewers. Any product that may be evaluated in this article, or claim that may be made by its manufacturer, is not guaranteed or endorsed by the publisher.
